# Adipose Tissue and Endocrine-Disrupting Chemicals: Does Sex Matter?

**DOI:** 10.3390/ijerph17249403

**Published:** 2020-12-15

**Authors:** Brigitte Le Magueresse-Battistoni

**Affiliations:** 1 Univ-Lyon, CarMeN Laboratory, INSERM U1060, INRAé U1397, Université Claude Bernard Lyon1, F-69310 Pierre-Bénite, France; brigitte.lemagueresse@inserm.fr; Tel.: +33-(0)-426235919; Fax: +33-(0)-426235916; 2 CarMeN Laboratory, INSERM U1060, Hopital Lyon-Sud, Bâtiment CENS ELI-2D, 165 Chemin du Grand Revoyet, 69310 Pierre-Bénite, France

**Keywords:** endocrine-disrupting chemicals, metabolic disorders, sex-dimorphism, estrogen, glucocorticoid, adipose tissue

## Abstract

Obesity and metabolic-related diseases, among which diabetes, are prominent public health challenges of the 21st century. It is now well acknowledged that pollutants are a part of the equation, especially endocrine-disrupting chemicals (EDCs) that interfere with the hormonal aspect. The aim of the review is to focus on adipose tissue, a central regulator of energy balance and metabolic homeostasis, and to highlight the significant differences in the endocrine and metabolic aspects of adipose tissue between males and females which likely underlie the differences of the response to exposure to EDCs between the sexes. Moreover, the study also presents an overview of several mechanisms of action by which pollutants could cause adipose tissue dysfunction. Indeed, a better understanding of the mechanism by which environmental chemicals target adipose tissue and cause metabolic disturbances, and how these mechanisms interact and sex specificities are essential for developing mitigating and sex-specific strategies against metabolic diseases of chemical origin. In particular, considering that a scenario without pollutant exposure is not a realistic option in our current societies, attenuating the deleterious effects of exposure to pollutants by acting on the gut-adipose tissue axis may constitute a new direction of research.

## 1. Introduction

Obesity and metabolic-related diseases, among which diabetes, are prominent public health challenges of the 21st century. According to WHO (World Health Organization, Geneva, Switzerland), obesity is “abnormal or excessive fat accumulation that presents a risk to health” and is diagnosed at a body mass index (BMI) ≥ 30 kg/m^2^ [1]. Overweight (BMI 25.0 to 29.9) and obesity lead to adverse metabolic effects on blood pressure, cholesterol, triglycerides, and insulin resistance and increased risk of co-morbidities. In 2016, close to 2 billion adults were overweight, of whom more than half a billion were obese (11% of men and 15% of women), and 340 million children and adolescents aged 5 to 19 were overweight or obese. Both developed and developing countries are concerned, particularly in urban settings. Metabolic diseases are marked by severe social and psychological difficulties that affect the overall quality of life, especially when obesity sets early in life. In addition, life expectancy is reduced because of increased mortality ranging from chronic non-communicable diseases including diabetes, cardiovascular disease to certain types of cancer [2]. Metabolic-related diseases also impose a heavy economic burden on societies. European Union countries and the United States of America spend approximately 7% and 21% of their health care budgets on obesity-related diseases [3,4], respectively. As the prevalence of obesity does not appear to be declining and the list of risks associated with obesity and diabetes has grown further with the COVID-19 infection [5], these diseases will continue to represent an increasing burden on society and health systems in the years to come.

Reducing the burden of obesity requires a better understanding of the multiple etiological causes interacting with each other whose effects can be or not age-, gender-, and ethnicity-dependent, not to mention wealth and social practices. Basically, excessive food intake and reduced energy expenditure cause energy imbalance. However, it is much more complex to understand the interweaving of the biological, environmental, and societal factors that can modify either food intake and/or energy expenditure. Based on the kinetics of the obesity pandemic that has paralleled industrialization, it has also been suggested that pollutants contributed to this phenomenon [6,7], for which was coined the term obesogen [8]. Indeed, industrialization has caused the release of thousands of anthropogenic molecules which resulted in the contamination of various environmental compartments, sometimes very distant from the production site, thus globalizing pollution. Many chemicals involved have primarily been identified as endocrine-disrupting chemicals, i.e., chemicals capable of altering any hormonal action [9,10,11]. Therefore, energy homeostasis that is largely under hormonal regulation, especially sex steroids [12] is at high risk of unbalance by endocrine disruptors in a sex-dependent manner. Accordingly, the prevalence of obesity and metabolic-related diseases differ in male and female individuals [13,14]. In women, the main contributors to metabolic syndrome are high body mass index (BMI) and hyperglycemia, while in men it is hypertension and high triglycerides [15].

The purpose of the review is to focus on adipose tissue because it is a central regulator of energy balance and metabolic homeostasis. Significant differences on the endocrine functions of the adipose tissue between males and females are highlighted, as well as the sex-specific dysfunctions in response to exposure to endocrine disruptors. Indeed, a better understanding of the mechanism by which environmental chemicals target adipose tissue and cause metabolic disturbances, how these mechanisms interact and the sex specificities is essential for developing mitigating and sex-specific strategies against chemical-induced metabolic diseases. In particular, the gut-adipose tissue axis may constitute a new direction of research.

## 2. Sex-Dimorphic Traits of the Adipose Tissues and Molecular Determinants

### 2.1. Body Fat and Distribution

Total body fat content and distribution are highly sex-dimorphic traits. Genome-wide association studies clearly established a genetic component to variations in fat distribution and obesity. White adipose tissues (WAT) comprise various depots such as inguinal (Ig), interscapular (Is), perigonadal (Pg), retroperitoneal (Rp), and mesenteric (Ms) depots, classified into subcutaneous (Ig, Is) and visceral (Pg, Rp, Ms) based on their anatomical location. In addition to white adipocytes, mammals have brown adipocytes which store less lipids and have more mitochondria than white adipocytes. In rodents, the brown adipose tissue (BAT) is mainly localized to the interscapular region during neonatal life and can be found in WAT depots following cold exposure [16]. Brown adipocytes are characterized by the expression of uncoupling proteins (UCPs) which allow dissipation of energy as heat. Beige adipocytes exhibit properties of both white and brown adipocytes (mitochondria and expression of UCPs) and are located preferentially in subcutaneous WAT depots in mice [17]. 

Women have a higher overall body fat content than men, particularly in the gluteofemoral depot (i.e., gynoid obesity). Men have higher overall muscle mass than women and will accumulate fat in a visceral and abdominal distribution (i.e., android obesity). After menopause, lack of circulating estrogens will cause a shift in fat accumulation and women will preferably accumulate visceral fat. From an evolutionary point of view, in women the accumulation of subcutaneous fat constitutes energy reserves to prepare for the development of pregnancy and breastfeeding. The metabolic differences between the sexes depend on the type of fat accumulated. Indeed, the accumulation of subcutaneous fat is not detrimental to metabolic health unlike the accumulation of visceral fat which predisposes to diabetes and cardiovascular disease. This explains that the menopausal transition puts women at equal risk to that of men for the development of metabolic syndrome [18]. The increased metabolic and cardiovascular risks associated with abdominal obesity could be explained by various differences in vascularity, innervation, and functions between the visceral (v) and the subcutaneous (sc) white adipose tissue (WAT) [19]. The latter is the physiological reservoir for excess fat as triglycerides (TGs) in response to caloric excess. It is characterized by its ability to generate new adipocytes (hyperplasia) sensitive to the anti-lipolytic effects of insulin and its large capacity to store fat as TGs and produce various adipokines such as adiponectin. Adiponectin is an adipokine with insulin-sensitizing properties acting in various metabolic organs (liver, skeletal muscle, brain) including the adipose tissue [20]. Unlike scWAT, the expandability of vWAT is limited. It contains large adipocytes sensitive to lipolysis with rather fewer small pre-adipocytes capable of differentiating into adipocytes. Release of free fatty acids (FFA) and lipid deposition in other tissues, in particular liver are processes known as lipotoxicity which triggers non-alcoholic fatty liver diseases (NAFLD) and insulin resistance. Altered expression of adipokines, synthesis of inflammatory proteins, and recruitment of pro-inflammatory macrophages are other characteristics which largely contribute to the enhanced pro-inflammatory state observed in obesity and diabetes [21]. 

### 2.2. Cell Lineage and Adipocyte Precursors

Adipose tissue has a mesodermal origin. Mesenchymal cells (MSCs) have extensively been used to model differentiating mesoderm into adipocytes, osteoblasts, or myocytes by applying appropriate stimulatory conditions. Signals inducing mesodermal and adipose development rely on several conserved developmental cues which include the bone morphogenetic proteins (BMPs), nodal, wingless (Wnt) and fibroblast growth factors (FGFs) [16], and on the retinoid X receptor (RXR) [22]. Pre-adipocyte differentiation (extensively studied using the mouse 3T3 cell line) is orchestrated by a transcriptional cascade in which the nuclear peroxisome proliferator-activated receptor (PPAR)γ which heterodimerizes with RXR, is essential to induce and maintain the differentiation of the adipocyte. The transcriptional cascade also involves members of the C/EBP family [16,17,23]. Interestingly, cell lineage tracing experiments in mice demonstrated distinct developmental origins depending on the adipocytes type and sex, which likely contribute to the functional sex- and depot- differences [24]. Gene expression profiling of adipose tissues identifies divergent molecular signatures between the various depot locations [25]. Nonetheless, markers capable of distinguishing MSCs from preadipocytes are still lacking and hamper the identification of preadipocytes with different developmental potentials and possibly, sex specificities. 

At birth, the testosterone surge in males plays a primary role in the masculinization of energy intake, feeding behavior, distribution of adipose tissue and adipocyte size, insulin sensitivity and adipokine secretion as shown experimentally with prolonged exposure of males to testosterone and neonatal exposure of females to androgens [12,26,27]. With the onset of puberty, estrogens and androgens are the major actors in the development of adipose tissue. In adults, use of transplantation methodologies showed that the microenvironment of visceral or subcutaneous fat depot (including the extracellular matrix and the cellular composition) could influence sex- and depot-dependent metabolic and growth (expandability) properties, in addition to the cell autonomous genetic differences [28,29,30]. In addition, while there is no evidence for sex differences in BAT distribution, female mice after puberty express higher levels of the uncoupling proteins (UCP) 1 and UCP3 than their male counterparts, indicative of positive regulation by estrogens [31]. 

### 2.3. Steroid Hormones and Direct Effects on Adipocyte Metabolism and Expansion

Sexual dimorphism of adipocyte metabolism and expansion largely depends on the mechanisms involving estrogen and androgen receptors. Estrogens enhance preadipocyte proliferation and differentiation into insulin-sensitive adipocytes and inhibit lipolysis, while androgens exert opposite functions [12,32,33]. However, the overall effect of estrogens and androgens is not simply due to the expression levels of receptors. Other parameters are involved such as the relative ratio of receptors expressed per depot, the local levels of active steroids, and the microenvironment status. 

#### 2.3.1. The Microenvironment Status and Extracellular Matrix (ECM) Remodeling

The adipose tissue microenvironment is made up of several types of cells including macrophages, immune cells, and fibroblasts in addition to preadipocytes and adipocytes. It is surrounded by a network of structural molecules such as different types of collagens and adhesion proteins forming the extracellular matrix (ECM) serving as the mechanical support. Adipose tissue expansion and regression occur throughout life and is associated with angiogenesis and the remodeling of ECM whose composition reflects a balance between matrix synthesis and degradation. Dysregulation of ECM proteolysis causing increased matrix deposits such as collagen 6a3 and altered expression of matrix metalloproteinases critical for ECM turnover [34,35], is a hallmark of fibrosis development and is tightly associated with inflammation and infiltration of macrophages in the adipose tissue [36]. 

Interestingly, most of the metalloproteinases (MMPs) and their tissue inhibitors (TIMPs) as well as the fibrinolytic system composed of plasminogen activators and inhibitors are expressed in adipose tissue and modulated with obesity [37], in a sex-dependent manner as shown for several MMPs (e.g., MMP-3 and -14), and TIMPs (e.g., TIMP-1, -2, and -4) [30,38,39]. Therefore, the adipocyte death and turnover mechanisms that support the expansion of fat depot may well function distinctly in males and females, especially in obesogenic nutritional conditions which are more deleterious in males than in females in terms of adipocyte death, formation of crown-like structures, inflammation, and fibrosis [40]. Angiogenesis and sympathetic innervation, which participate in the regulation of metabolism and fat expansion, could also be regulated differently depending on sex and explain the better metabolic health outcome in women compared to men, in response to an obesogenic environment [30]. 

#### 2.3.2. Repertoire of the Estrogen and Androgen Receptors in the Adipose Tissue

Estrogen and androgen receptors are expressed in the adipose tissues of both sexes but levels of expression can present sex-, fat depot- and species- specificities. For example, and as seen in rodent adipose tissues, human subcutaneous and visceral adipose tissues express both ERα and ERβ, whereas only ERα has been identified in pre-adipocytes [41] and in brown adipose tissue [42]. In addition, the adipose tissues in females showed higher levels of both ERα and ERβ than in males, and the relative level of expression of ERα to ERβ differs with sex between fat depots during differentiation steps. These features are probably important in the sexual-dimorphism of the adipose tissue metabolism, cellularity, and expansion. It is consistent with the intrinsic differences in the capacity of adipose tissue to respond to estrogens and with the regional effects of estrogens in the upper versus lower body adipose tissues of overweight to obese premenopausal women [32,43]. Indeed, ERα/ERβ ratio is higher in the adipose tissue of pre-menopausal women compared to postmenopausal women. In post-menopausal women, improvements of insulin sensitivity by exogenous estrogens was associated with an increase of the ERα/ERβ ratio in the subcutaneous adipose tissue [44]. Thus, it has been hypothesized that the higher ERα/ERβ ratio observed in the abdominal visceral fat of women may serve to limit adipose accumulation in this depot while the lower ERα/ERβ ratio in gluteal fat would provide a more favorable environment for adipose accumulation and storage in premenopausal women [18,32]. Contribution of the membranous G-protein-coupled estrogen receptor (GPER) in the adipose tissue remains to be defined but it may be marginal considering that no-sex or depot-specific *Gper1* expression has been demonstrated in mice [45,46]. Distribution of androgen receptors (ARs) in humans differed in preadipocytes and adipocytes based on fat depots. No sexual-dimorphism was demonstrated but the levels of ARs expressed in the visceral adipose tissue is two-fold higher than in subcutaneous fat depots indicating a higher effect of androgens on vWAT than on scWAT in women and men [47]. In mice, AR expression is both depot and sex specific. In females, AR mRNA levels are 2.5 times higher in vWAT compared to scWAT as seen in male vWAT and scWAT (BLM, unpublished data). Therefore, depending on species, the relative level of estrogen to androgen receptors may also contribute to the sex-dimorphism of the adipose tissue metabolism, cellularity, and expansion.

#### 2.3.3. Metabolic Function of the Sex Steroid Receptors in the Adipose Tissue

Generation of mice specifically devoid of ERα in adipocytes demonstrated its role in the regulation of adipocyte size. Lower levels of ERα resulted in enlarged adipocytes, adipose tissue inflammation, and fibrosis in both sexes [48]. It highlighted the role of adipocyte ERα in the phenotype of global ERα^-/-^ male and female mice which showed increased body weight and fat mass, insulin resistance, and impaired glucose tolerance [49] (Table 1). The function of ERβ is less clear. On the one hand, ERβ KO male mice fed a standard diet did not show changes in body weight or fat mass compared to wild-type mice. This condition was not studied in females [50]. On the other hand, the loss of ERβ in female mice (fed on a high-fat diet) caused an increase in PPARγ activity and in sensitivity to insulin in adipocytes, suggesting that ERβ can oppose PPARγ and thus can exert a pro-diabetogenic action in wild-type female mice fed a high-fat diet [51]. Unfortunately, this study did not examine the potential compensatory involvement of ERα or GPER in the ERβ KO mice. Indeed, other studies showed that ERβ agonists caused reduced body weight, fat mass and leptin and glucose parameters in ovariectomized mice [52], and browning of subcutaneous abdominal fat in obese female mice [53]. In addition, gonadal fat pads of both sexes showed enhanced levels of ERβ in mice specifically devoid of ERα in adipocytes. In the study of Davis and colleagues [48], the authors showed that knockdown of ERβ in adipocyte-specific ERα^-/-^ of both sexes did not cause further changes in total body weight but enhanced expression of fibrosis markers and inflammation in the adipose tissues of both sexes. Females in this study [48], also showed worsening glucose intolerance despite no change in plasma levels of 17β-estradiol. No change in glucose tolerance was noted in males. Altogether, it indicated a protective role of ERβ against fibrosis and inflammation when ERα is lacking. It also highlighted that ERβ is more important in females than in males. The precise role of ERβ in the adipose tissue awaits further studies.

As for the estrogens, the local action of androgens differ from its global metabolic effect. On the one hand, metabolic dysfunction and predisposition to diabetes is observed in males with androgen deficiency and in females with hyperandrogenism as shown with the polycystic ovary syndrome (PCOS), a very common disorder in women of childbearing age with a prevalence of 6–15%. Insulin resistance is a fundamental pathogenic metabolic component in PCOS in addition to the high prevalence of abdominal obesity, type 2 diabetes, and metabolic syndrome [54]. On the other hand, direct effects of androgens on adipocyte proliferation and differentiation are inhibitory [33]. In the same way, global AR knockout strategy or hepatic-, muscle-, or pancreas-specific KO results in male obesity with decreased energy expenditure, hepatic steatosis, and insulin resistance [55]; but, male mice lacking AR in adipose tissue do not exhibit obesity or enhanced fatness probably due to increased estradiol levels measured in adipose tissue [56] (Table 1). Unfortunately, the metabolic phenotype of female mice lacking AR in adipose tissue has not been reported. It is possible that no effects would be seen because the female adipose tissue is already under estrogenic regulation. Demonstration that GPER can induce sex-specific effects will await the generation of mice lacking GPER specifically from adipocytes. However, since *Gper1* expression in mice showed no difference between fat depots and sex [45,46], this hypothesis seems unlikely. 

#### 2.3.4. Local Production of Active Sex Steroids in the Adipose Tissue

Production of locally active steroids involves a large repertoire of enzymes that induce, interconvert, and inactivate peripheral sex steroid hormones. In both sexes, adipocytes of the visceral depot (predominantly) contribute to local production of active sex steroids by aromatization of the adrenal androstenedione and gonadal testosterone [18]. The metabolic benefit of aromatization is well illustrated in both knockout aromatase (ArKO) mice and in mice overexpressing aromatase in adipocytes. ArKO mice of both sexes grow larger with aging than age-matched wild-type mice and exhibit dramatic expansion of the visceral depot with larger adipocytes [57] while aromatase overexpression in adipocytes led to reduced inflammation and increased insulin sensitivity of adipose tissue in males [32] (Table 1). It would be interesting to study female mice overexpressing aromatase (not performed in Bracht et al. [32]). Indeed, insulin resistance is observed in women when circulating estrogen levels reach supra-physiological levels [58]. 

The steroid sulfatase (STS), a desulfating enzyme that converts steroid sulfates to hormonally active steroids and the estrogen sulfotransferase (EST) which inactivates estrogens play important roles in the homeostasis of sex hormones, in the adipose tissues (in addition to the liver). EST expression in WAT is testosterone dependent which is consistent with the high levels detected in male but not female WAT. Consistently, whole body deletion of EST in females did not affect the adipose tissues. On the contrary, males exhibited highly dysfunctional adipocytes characterized by elevated expression of several macrophage markers and a high density of crown-like structures, all signs of a local inflammation which could explain the worsening of metabolic abnormalities under obesity conditions [59] (Table 1). Nonetheless, differences exist in the regulation and function of EST between humans and mice. In humans, EST is detectable in the subcutaneous adipose tissue in both obese men and women, especially in the abdominal area, and it is significantly associated with markers of inflammation such as tumor necrosis factor (TNF) α [60]. 

STS is expressed in the adipose tissue of both males and females and levels of expression are increased in obese mice. Interestingly, transgenic overexpression of the human STS in the adipose tissue produced sex-dimorphic effects in mice fed a high-fat diet. In females, STS transgene expression alleviated metabolic functions and decreased inflammation in the adipose tissue. Unlike females, males exhibited worsened metabolic health, including weight and fat gain, as well as aggravating insulin resistance, which was due to enlarged adipocytes, increased fat inflammation, and dysregulated adipogenesis. These effects were related to increased androgenic activity since castration abolished the effects [61] (Table 1). 

Adipose tissue is also capable of active androgen synthesis through the aldo-keto reductase 1C (AKR1C) or 17β-hydroxysteroid dehydrogenase (17β-HSD) family of enzymes. One of the three isoforms of 5α-reductase, the SRD5A1 is expressed in adipose tissue and may contribute to androgen production of fat, as reviewed elsewhere [33]. Glucocorticoids are powerful regulators of adipose tissue metabolism. Produced by the adrenal glands, they influence body fat distribution and interact both with estrogens and androgens. In the adipose tissue, glucocorticoids promote adipogenesis and preadipocyte differentiation, through the regulation of the 11β-hydroxysteroid dehydrogenase 1 (11βHSD1). This enzyme catalyzes the conversion of inactive cortisone into active corticosterone allowing local amplification of glucocorticoid action in the absence of plasma corticosterone levels variations [62,63]. Consistently, transgenic overexpression of 11β-HSD1 in adipocytes lead to GC-induced hypertrophy of visceral adipose tissue [64] and its specific deletion in adipocytes of male mice improves metabolic profile and resistance to diet-induced obesity. Adipose tissue hypertrophy and secretion of inflammatory markers are reduced while insulin sensitization is improved [65]. Interestingly, expression of 11β-HSD1 is higher in scWAT than vWAT of male or either WAT of female [46]. This is consistent with the findings that the glucocorticoid-induced insulin resistance is more severe in male than in female mice [66]. Finally, and in contrast to the liver where GH plays a central role in sexual dimorphism [67,68], GH plays a similar role in the adipose tissue of both sexes. This was evidenced using adipose-specific GH receptor-KO (GHRKO) mice where GH opposed scWAT expansion to a similar trend in both sexes [69] (Table 1).

## 3. Overview of Metabolic Disruptors and EDCs Targeting the Adipose Tissue

The term metabolic disruptors has been proposed by Casals-Casas and Desvergne [70] to design chemicals and their metabolites whose detection in blood or urine significantly correlated with the criteria of metabolic syndrome (obesity, dyslipidemia, hypertension…). This denomination was based on several epidemiological studies, some of which included a large number of individuals [71,72,73] as well as from experimental studies on rodent models, which demonstrated strong relationships between chemical exposure and obesity and metabolic-related diseases [74,75,76,77,78,79]. Several of these metabolic disruptors work by interfering with hormonal actions and hence are referred to endocrine-disrupting chemicals (EDCs) [9].

EDCs have several modes of action. EDCs can bind to hormone receptors and exert agonist or antagonist activities modifying the downstream signaling cascade. They can interact indirectly with hormone receptors by enhancing or suppressing the response to endogenous hormones. They can also modulate endogenous hormone levels by acting on enzymes that induce, interconvert, and inactivate peripheral sex steroid hormones, particularly expressed in the adipose tissue as reviewed above. Several characteristics of EDCs further complicate the issue. EDCs can act at very low doses and in a non-monotonous manner, challenging the principles of toxicology which are based on the concept of linearity of harmful effects of chemicals above a threshold value. Calculations are done empirical, i.e., a linear dose-response curve is assumed from the dose at which no or low adverse effects (N(L)OAELs) are observed in animal studies. From then, a tolerable daily intake (TDI) can be extracted, which is the estimate of the amount of chemical in food or drinking water that can be ingested daily for a lifetime without significant risk to health [80]. Therefore, toxicological reference values based on (N(L)OAELs) established by international agencies such as the United States Environmental Protection Agency (EPA) or the European Food Safety Agency (EFSA) may not be sufficiently protective for some EDCs likely to act at concentrations estimated to be safe for the environment as shown with bisphenol A (BPA) [81]. 

Another important feature is that endocrine disruptors, by definition, will have a greater effect during times of high endocrine changes and can display long-term effects. For example when exposure has taken place during fetal and/or neonatal periods, effects can be observed in adulthood. This is known as the developmental origins of health and diseases (DOHaD) [82,83]. Puberty, menopausal, and post-menopausal periods are also periods of important endocrine changes, therefore sensitive to the potential deleterious effects of EDCs. Finally, it should be considered that humans are unintentionally exposed to a plethora of low levels of chemicals during lifetime that could interact with each other resulting in effects distinct from those exerted individually. These complex effects are known as cocktail effects and have been demonstrated experimentally with natural or reconstituted mixtures of compounds [75,84,85,86,87,88].

To date, around 1000 EDCs both synthetic and natural chemicals have been categorized as endocrine-disrupting chemicals [89]. Exposure can occur by inhalation or contact, but food is the primary route of exposure for many EDCs. EDCs include metals, industrial chemicals, personal and household care products (e.g., alkylphenol, phthalates, parabens, musk and flame retardants compounds), pesticides, herbicides, fungicides, pharmaceutical drugs, and synthetic or naturally occurring hormones [9,11,90]. Persistent organic pollutants (POPs) include highly lipophilic industrial compounds with low-degradability, such as dioxins, polychlorinated biphenyls (PCBs) and polybrominated flame retardants, or organochlorine pesticides such as dichlorodiphenyltrichloroethane (DDT) and organometallic compounds such as tributyltin (TBT). Dioxins are unintended by-products resulting from incomplete industrial combustions (but also during forest fires or volcanic eruptions). They constitute a large family of which 2,3,7,8-tetrachlorodibenzo-p-dioxin (TCDD) is the most potent. Consistently with the definition of an EDC, evidences show a non-linear relationship between dioxin exposure and body weight [91]. At low dosage, TCDD exposure during adulthood enhanced body weight [92], while high and acute TCDD exposure caused a wasting syndrome characterized by severe weight loss accompanied by a decrease in adipose tissue mass in rodents, although toxicity differed among species (i.e., sensitive *versus* resistant strains) [93]. Periods of exposure are also important. For example, low dose TCDD exposure caused obesity in male and female mice exposed in adulthood [94] but not after perinatal exposure [95]. Dioxins mediate their actions by activating the aryl hydrocarbon receptor (AhR) which is a multifaceted ubiquitously expressed receptor originally categorized as an orphan receptor [96], and deeply involved in the regulation of energy homeostasis as evidenced by genetic manipulation. Over-activation of AhR was shown to promote obesity, hepatic steatosis, nonalcoholic steatohepatitis, and insulin resistance [97,98]. Consistently, mice deficient for AhR are resistant to diet-induced body weight gain, steatosis, and inflammation through increased energy expenditure [99]. The same phenotype of resistance to diet-induced obesity was described in mice with specific deletion of AhR in pre-adipocytes through the expression of Pdgfrα-Cre [100]. In contrast, AhR deficiency in mature adipocytes through expression of adiponectin-Cre [101] resulted in obesity, enhanced fat mass, and larger visceral adipocytes, a phenotype which was exacerbated in response to a high-fat diet. These studies while highlighting the involvement of adipocyte AhR in the regulation of adiposity, adipose inflammation, body weight, and glucose homeostasis in mice suggest that in males (no data are yet available for females), the timing of AhR knockout (i.e., at the pre-adipocyte or differentiated adipocyte stage) during the course of adipogenesis may be important for determining the outcome. 

In addition to dioxins, AhR can be activated by polycyclic aromatic hydrocarbons (PAH) found in cigarette smoke, wood burning, and overcooked meat and dioxin-like PCBs, i.e., coplanar PCBs. PCBs were designed for their low-flammability and high thermal conductivity properties. The adverse metabolic effects of PCBs may also be mediated by the interaction with estrogen and thyroid signaling pathways, especially in the case of non-coplanar PCBs also called the non-dioxin-like (NDL) PCBs as they do not bind AhR [11,70,102]. Another mechanism may involve the binding of PCBs to the xenoreceptors, constitutive androstane receptor (CAR), and pregnane X receptor (PXR) mostly expressed in gut and liver [103], and which could secondarily affect adipose tissue [104,105]. Because of their toxicity, PCBs have been replaced by brominated flame retardants, which are added to manufactured materials, such as plastics, textiles, surface finishes, and coatings to prevent or slow the further development of ignition. Yet, these chemicals were shown to interfere with PPARγ and thyroid signaling, and are as well classified as EDCs [11,70,106]. For example, perinatal exposure to decabromodiphenyl ethane (DBDPE) [107] or adult exposure to hexabromocyclododecane (HBCD) [108] resulted in enhanced body weight and metabolic dysfunctions in male mice (females were not studied). 

DDT is a powerful insecticide once widely used to control insects in agriculture and insects that carry diseases such as malaria. It was banned in 1972 in the United States because of its damage on wildlife. Both DDT and its metabolite dichlorodiphenyldichloroethylene (DDE) are presumed obesogens in humans [109]. TBT is a powerful biocide. It is used in marine shipping applications, as fungicides for paper mills and for industrial treatment of water. TBT activates both PPARγ and RXR inducing differentiation of adipocytes [110,111,112] but not all the health-promoting activities induced by PPARγ and in particular not the pathways linked to mitochondrial biogenesis or those involved in beiging [113]. It elicited transgenerational effects through epigenetic mechanisms not yet fully characterized, and early life exposure of mice to TBT caused reprogramming of adipocyte stem cells and promotion of adipocyte lineage into subsequent unexposed male and female generations [112,114]. Mechanistic studies showed that TBT can promote adipose lineage commitment of undifferentiated mesenchymal stem cells (MSCs) through the activation of RXR but not PPARγ, reprogramming stem cell fate to a dysfunctional adipocyte, and highlighting the deleterious role of RXR-disrupting chemicals on adipose tissue development and function [115]. Dibutyltin (DBT), the major breakdown product of TBT which also activates both RXR and PPARγ leads to predisposition to obesity, as well [112]. 

Concerns were also raised about other industrial chemicals such as bisphenols and phthalates. They are not persistent in the environment but they are massively used in the plastic products found in many aspects of modern life. Therefore, they are ubiquitous in the environment and can leach from food and beverage containers and packaging to cause contamination [81,116] resulting in chronic exposure. Phthalates and bisphenols target adipose tissue. Despite structural similarities, these large families of chemicals exhibit distinct obesogenic and/or diabetogenic potential. For example, BPA which is the most emblematic endocrine disruptor [117,118,119,120] has initially been described as pro-estrogenic because it binds to estrogen receptors. In vivo, rodent studies show that BPA can increase metabolic disturbances within insulin-sensitive organs including the adipose tissue eventually leading to type 2 diabetes without systematically causing gain of weight [121,122]. Studies done in 3T3-L1 cell line model showed that BPA promotes adipogenesis through mechanisms that do not necessarily depend on the activation of PPARγ [121,123,124,125,126]. Depending on the congener considered, the phthalates induced distinct metabolic adverse effects which could depend on the distinct binding affinities of the congeners toward PPAR. For example, butyl benzyl phthalate (BBP) and the diethyl-hexylphthalate (DEHP) metabolite MEHP could activate PPARγ causing enhanced lipid accumulation [125,127,128,129]. Additional modes of action of BPA may include activation of the glucocorticoid receptor (GR) as shown with phthalates [130] and interaction with thyroid signaling [131]. In addition, both BPA and phthalates can exert anti-androgenic activities although phthalates unlike BPA do not bind to the androgen receptor [119,132]. BPA was banned for the manufacture of polycarbonate infant feeding bottles by regulatory agencies in the United States, Canada and European Union in the 2010s. It prompted the plastics industry to develop BPA analogs whose safety is a matter of debate because of their structural similarities with BPA. For example, Bisphenol S (BPS) which is particularly used to replace BPA in the production of epoxy resins, thermal papers, and infant feeding bottles was also shown to cause metabolic adverse effects [133]. Cell line models (3T3-L1 cells) showed that BPS could enhance lipid accumulation possibly through PPARγ- [134] or ERRγ- [135] dependent mechanisms and caused increase in body weight in male mice fed a high-fat high-sugar-rich diet but not in sibling females [136]. As for BPA, BPS can efficiently activate both human (h) ERs but is a weak agonist of hAR [137]. 

Large-scale initiatives were undertaken to identify which chemicals had obesogenic properties based on the activation of PPARγ, the master regulator of adipogenesis. Experiments were carried out using various models of adipocyte cell lines such as the cell line murine 3T3-L1, primary cultures of fat depots recovered from animal or human donors, and transfected cells coupled to gene reporter assays. Chemicals leading to lipid accumulation included organophosphorous pesticides [138,139], imidazole fungicide triflumizole [140], various flame retardants [141,142,143,144], parabens, and musk compounds [125,145], DDT/DDE [146,147]. As for bisphenols and phthalates, the activation of PPARγ has not been systematically reported suggesting the involvement of other mechanisms to induce lipid accumulation. For example, flame retardant tetrabromobisphenol A (TBBPA), may interact with GR and ER signaling mechanisms in addition to PPARγ [143], and house dust extracts that contained flame retardants have been shown to act as thyroid receptor antagonists [148]. A synthetic table (Table 2) recapitulates most of the findings of this section.

Metabolic disruptors can also induce mechanisms that are not strictly related to hormonal action including mitochondrial dysfunctions and oxidative changes [148], alterations in the expression of circadian clock genes [46,149], epigenetic mechanisms, and gut microbiota modifications [112,150,151,152], but also alterations of RXR signaling as evidenced with TBT [115] and probably with other chemicals [153]. RXR is unique in that it forms heterodimers with different nuclear receptors defined as a permissive or non-permissive partner. With a permissive partner, each of the two receptors of the heterodimer can be activated by its own ligand potentially leading to a response of a greater amplitude than that resulting from the binding of a single ligand to its receptor. Within the adipose tissue, permissive receptor partners include PPARs (PPARα, β/δ and γ), liver X receptors (LXRs), and farnesoid X receptors (FXRs) whose ligands include fatty acids, cholesterol metabolites, and bile acids, respectively which provides to RXR activators a mechanism to control transcription of a large set of genes [154]. The potential modifications of the microbiota induced by exposure to pollutants, in particular those which could induce modifications of the intestine-adipose axis or would result from the modifications of this same axis are presented in the last part of this review.

## 4. The Adipose Tissue Is Both a Storage and a Primary Target of EDCs. Are There any Differences between Male and Female Individuals?

The Stockholm convention of 2001 (and subsequent implementations) strictly regulated the production of POPs because of the acute toxic effects on humans and ecosystems, banning the production of DDT and PCBs and limiting the production of dioxins, among others. However, POPs are highly lipophilic compounds with low degradability, and they continue to contaminate soil, sediment, and food through biomagnification in the food chain, especially fatty foods which are the main route of exposure. Consequently, persistent pollutants concentrate in the adipose tissues of animals and humans where they can be stored for years [155]; even though, the decline in the use and production of PCBs and of organochlorine pesticides, to cite only two, was correlated with a decrease in concentration in the samples mentioned above [156,157]. Therefore, it has been hypothesized that the POPs stored in AT could be the key to the pathogenesis linking impaired AT function and metabolic-related diseases especially promoting inflammation [97,158], bearing in mind that vWAT and scWAT do not have the same endocrine functions, and that POPs depending on family or on congeners may show distinct deleterious effects. In the context of this review, an important question was whether the distribution of POPs in fat depots and their release from fat depots toward the bloodstream and their effects on metabolic organs could differ by sex, and how this would translate in terms of metabolic and endocrine disturbances.

There is yet no consensus on whether POPs distribute evenly in fatty compartments. The differences may be related to cohorts showing a distinct history of exposure to EDCs because of lifestyle, eating habits, and geographic location. Genetic differences in metabolic rates and body mass index can also contribute. In women, the number of pregnancies and whether the children were breastfed or not can influence the levels of POP accumulation [159]. Thus, dissimilar POPs storage capabilities in WAT depot have been described in a Portuguese cohort of obese patients in which vWAT showed higher levels of Σ POPs than scWAT, but differences between men and women were not evaluated [160]. Heterogeneity has also been reported with respect to the association of individual POPs with WAT mass [161,162,163]. Other studies could not conclude on a preferential distribution of Σ POPs in vWAT over scWAT [164,165,166] or in men compared to women [164,166]. Moreover, POPs concentrations have not been found significantly different between subgroups of normal-weight, overweight, or obese men and women [166].

Since vWAT is a highly metabolic active tissue more susceptible to lipolysis than scWAT, one could hypothesize that vWAT (more than scWAT) released POPs (or certain POPs) into blood supporting the hypothesis that scWAT (more than vWAT) may exert some protective effect by sequestering pollutants and preventing their systemic circulation [165]. It would be consistent with the beneficial role of scWAT expansion. An elegant xenografted fat model [167], showed that TCDD sequestered in the epididymal adipose tissues of donor mice could be released into the host to target metabolic organs. The host liver showed activation of AhR signaling pathways, disruption of carbohydrate and lipid metabolism, and increased inflammation and liver fibrosis. Thus, chemicals accumulated in vWAT can disrupt liver homeostasis [167]. The design of models of xenografted fat using scWAT explants may help determining if POPs release evenly from vWAT and scWAT stores. Obesity status should be investigated as well as the sex of the donor to explore any contribution of estrogen (also using ovariectomized host mice). Indeed, the effects caused by the accumulation of pollutants could be different in males and females, especially as the dioxin and estrogen signaling pathways extensively interact resulting in dioxins altering estrogen-regulated genes and in modulation of AhR activity by ERα [168,169,170]. Consistently, female mice exposed perinatally to TCDD (gestation and lactation) showed increased visceral fat pad weights (no data are available regarding scWAT) at adulthood while opposite findings were described in the sibling males [95]. Similar data have been reported in mice exposed perinatally to DL-PCB126 [171] but not to NDL-PCB153 [95] consistent with cross-interactions of the AhR and ER signaling pathways in the metabolic programming of energy homeostasis during gestation and lactation. TCDD exposure at adulthood also resulted in sex-dependent changes in body composition with enhanced fat mass in females but decreased visceral fat pads in males [94].

Unlike the liver or intestine, the adipose tissue is not a detoxifying organ per se. It does not express the constitutive androsterone receptor (CAR) or the pregnane xenobiotic receptor (PXR) which are major xenobiotic receptors in the liver and gut [172]. However, adipose tissues express several nuclear receptors [173] such as the estrogen and androgen, and glucocorticoid receptors but also PPARγ and RXR which can bind various xenobiotics with distinct affinities. As such, the adipose tissue is a primary target of EDCs, causing inflammation, mitochondrial dysfunction, and triggering insulin resistance potentially disrupting developmental programming [73,174], ultimately leading to metabolic-related diseases. In addition, adipose tissue can be indirectly targeted as shown in the case of CAR. Indeed, exposure to a mixture of low-dosed pesticides caused enhanced body weight and fat mass in addition to glucose intolerance and hepatic steatosis in wild-type but not in CAR^-/-^ male mice. These effects were not observed in wild-type females which suggests that CAR displays sex dimorphic effects [86]. Another study involving both wild-type and CAR^-/-^ male mice showed that exposure to PCB153 caused CAR-driven alterations in retinoid homeostasis in both the liver and adipose tissue negatively affecting the transcription of genes involved in lipid and carbohydrate metabolism in these tissues [175]. Interestingly, retinoids can activate RXR and CAR is a permissive partner of RXR [154]. It is not known if these effects are also present in females as they were not studied [175]. Whether the liver was the primary target organ with adipose tissue secondary effects awaits the generation of mice with specific CAR deletion in the liver.

Interestingly, Pestana and colleagues [160] reported a positive correlation between POPs content in vWAT and the presence of MetS, (especially dysglycaemia and hypertension) and cardiovascular risk, and with HOMA-IR, an index of insulin resistance for POP content in scWAT. Using a different cohort of obese patients, highly chlorinated PCBs and PDBEs were reported to be significantly and positively correlated with the measurement of abdominal adiposity which was fully attributed to a positive correlation with the vWAT and/or vWAT/scWAT ratio [164]. Within the same cohort, obesity markers (leptin, adiponectin and TNFα) correlated with the levels of certain POPs in vWAT but not in scWAT suggesting that vWAT could be more sensitive to the deleterious effect of POPs than scWAT. In addition, women but not men showed significant association between serum leptin concentrations and the levels of several PCBs in the two fat depots suggesting that the sensitivity to the obesogenic actions of POPs could be sexually biased [176]. While POPs could contribute to the aggravation of metabolic effects triggered by a hypercaloric diet [177,178], a recent study [166] using a cohort of normal-weight, overweight, and obese men and women exemplified that POPs abundance in WAT did not correlate with obesity but with adipocyte hypertrophy, macrophage infiltration, systemic inflammation, and impaired glucose metabolism. The effects were generally more pronounced in women than in men subjects. The authors concluded that bioaccumulation of POPs in WAT has a negative effect on WAT function especially in lean women [166]. 

## 5. EDCs Mimicking Sex Steroid or Altering Active Sex Steroid Levels in the Adipose Tissues and Differences of the Effects in Males Versus Females

The very first experiment demonstrating a link between obesity and increased fat mass and visceral adiposity in response to a synthetic estrogen was performed in female mice exposed to diethylstilbestrol (DES) during the neonatal period. Meanwhile, the male siblings experienced decreased body weight. The origin of the sexual dimorphism was not explored and only body weight has been recorded in the study [179]. DES is a synthetic estrogen administered to women between the 1940s and 1980s, to prevent miscarriages. Its use was prohibited because it led to an increased prevalence of very rare carcinomas in girls [180]. Thus, the experimental study of Newbold and colleagues [179] showed that the neonatal period is very sensitive to sexual hormones. Indeed, the first two weeks of neonatal life in mice, which corresponds to the last trimester of gestation in humans, is a period of developmental plasticity of the hypothalamic circuits controlling adiposity and the development of peripheral adipose tissue [16]. Consistently, perinatal exposure to DDT which is known to interact with sex steroid receptors also caused impaired metabolic homeostasis in female but not male mice. Specifically, female mice showed a transient early-life increased in body weight caused by BAT dysfunction and decreased energy expenditure leading to insulin resistance and the metabolic syndrome in adult mice fed a high-fat diet [181]. Another study showed that neonatal exposure of female mice to testosterone resulted in a masculinized pattern of fat accumulation with increased visceral adiposity and enlarged adipocytes and many of the features observed in women with PCOS [54]. In parallel, neonatal treatment of males with androgens showed reduced lean mass and food intake but enhanced subcutaneous adiposity and other characteristics of central hypogonadism with low testosterone levels [27]. It indicates that exposure to chemicals with estrogen and/or androgen activities during the period of adipose tissue development can reprogram predisposition to obesity in both sexes, although stronger effects would be detected in males for most outcomes [182]. Interestingly, perinatal exposure to BPA caused enhanced fat weight gain and disruption of weight control mechanisms [183,184] as well as reproductive and endocrine alterations resembling the PCOS syndrome in adult rats with hyperandrogenia [185]. Therefore, it has been suggested that endocrine disruptors especially BPA could be etiologic factors of PCOS syndrome in genetically predisposed individuals [186,187]. 

Nuclear steroid receptors originate from a common ancestral gene, which underlies the substantial homology in DNA-binding domain and hormone-responsive elements [188]. For example, GR shares numerous binding sites with AR [189] and a GRE element is located in the adipose-specific promoter of the aromatase P450 gene [190] highlighting complex cross-interactions between sex steroids and glucocorticoids in the regulation of energy homeostasis, especially in the adipose tissue which express glucocorticoid-metabolizing enzymes. It exemplifies why in women excess exposure to either androgens or glucocorticoids triggers adiposity and insulin resistance as described in women with PCOS or the Cushing syndrome, respectively. Interestingly, both estrogens and glucocorticoids share functions in the immune system as they are both anti-inflammatory molecules. Indeed, the metabolic and immune systems are closely associated systems in which adipose tissue is the source of inflammatory responses and mediators [191]. Thus, EDCs acting on GR or on corticoid metabolism through alteration of metabolizing enzyme expression such as 11β-HSD1 could be etiologic factors of the epidemic obesity and metabolic-related disorders. Tolylfluanind (TF) is a phenylsulfamide fungicide used in agriculture and as a booster biocide in marine paints. Because it is lipophilic, it may accumulate in the adipose tissue. In vitro experiments using the 3T3-L1 cell culture model demonstrated that TF had GR-agonist activities [130]. Moreover, perinatal exposure to TF showed sex-specific adverse effects on the adipose tissue [192] which may have epigenetic basis [193] at least in males (females had not been studied). Indeed, adult males had impaired glucose tolerance but no changes of adiposity if exposed perinatally while they showed enhanced body weight, adiposity, and insulin resistance if exposed during adulthood [194]. Consistent with the distinct regulation of the adipose tissue by glucocorticoids in males and females, female mice exposed perinatally to TF showed enhanced systemic insulin sensitivity, reduced adiposity and normal hepatic gluconeogenesis [193]. However, others demonstrated that TF impacted mitochondrial metabolism but failed to demonstrate obesogenic effects of TF [195]. Completion of the studies awaits dosage of the glucocorticoid (GR) and mineralocorticoid (MR) receptors as well of 11β-HSD1 which allows local amplification of GCs in the absence of changes in plasma corticosterone levels [62]. Indeed, it was shown that exposure of 5- to 20-week-old male and female mice to a low dose mixture of BPA, DEHP, PCB153, and TCDD affected the expression levels of GR, MR, and 11β-HSD1 in WAT according to sex and it was associated in females with clock-related gene modifications in the absence of changes in plasma corticosterone levels [46]. Clearly, understanding the underlying molecular basis for the sexually dimorphic actions of glucocorticoids in the adipose tissue is important, since synthetic glucocorticoids are the most widely prescribed medication for the treatment of chronic inflammatory diseases and hematological cancers in humans [196]. These findings also emphasize the pro-inflammatory effects trigger by certain EDCs [158,197] or mixtures of EDCs. For example, enhanced expression of inflammatory markers (Il6, Il1b, and Ccl5) in the WAT of adult females was shown after a lifelong exposure, including the fetal and lactation periods, to a mixture of chemicals comprised of TCDD, PCB153, BPA, and DEHP. No such effects were found in male mice [198]. 

Finally, although not yet explored, it is possible that the fate of adipocyte precursors in early-life exposure situations, the microenvironnement, and ECM remodeling of the adipose tissue depots throughout development and expansion as well as the local production of sex steroids are modified in response to EDCs, thus contributing to metabolic disturbances with potential sex specificities. Analyzing these aspects should help at identifying potential new markers of exposure to EDCs and at better defining how exposure to EDCs leads to deleterious effects.

## 6. Exposure to EDCs and the Gut-Adipose Tissue Axis

The gut microbiota plays major functions in host metabolism and immunity, digestive absorption and nutrient uptake, synthesis of vitamins and prevention of colonization of pathogens through the production of bacterial metabolites which can act in distant organs such as the brain, liver, and also the adipose tissue and which will be involved in host–microbiota interactions [199]. Obesity and insulin resistance in humans and rodents are associated with intestinal dysbiosis which is characterized by the enrichment of certain species of bacteria to the detriment of others (e.g., a change in the ratio of *Firmicutes* and *Bacteroidetes* which are the two dominant phyla representing more than 90% of the total community) with changes in the composition of the gut microbiota [200,201]. The gut-adipose axis depends on communication through short-chain fatty acids (SCFAs) and bile acids (BAs) which behave as hormone-like products signaling through G protein-coupled receptors (GPRs) expressed in the adipose tissue [202,203]. SCFAs which include acetate, propionate, and butyrate are the major end-products of microbial fermentation of dietary fiber. They serve as the primary energy source for colonic epithelium and deficiency in SCFA production is associated with type 2 diabetes. SCFAs communicate with the adipose tissue through GPR43 and GPR41 to promote energy metabolism, insulin sensitivity, glucose tolerance, and adipokine secretion [202,203]. BAs are the end-products of cholesterol which exert among others, metabolically beneficial functions in the adipose tissue through promoting beiging and enhancing thermogenesis upon activation of the bile acid-responsive G-protein-coupled receptor TGR5 [204].

Interestingly, evidences have accumulated that BPA, phthalates, TBT, PCBs, and dioxins among others can lead to gut microbiota disruptions through changes in the ratio of *Firmicutes* and *Bacteroidetes*, reduced diversity of the microbial communities and modulation of gut microbiota composition [205,206,207,208,209,210]. In addition, in several circumstances, significant associations were found between changes in gut microbiota, gut metabolite profiles, and metabolic outcomes such as obesity and dyslipidemia in mice exposed to chemicals [205,207,208,209,210,211,212]. One study identified that gut dysbiosis preceded the obesity phenotype shown in mice exposed to BPA [206]. Another study showed that prenatal exposure to DEHP resulted in enhanced body weight and reduced energy expenditure in the male offspring only along with gut dysbiosis [213]. Since environmental pollutants and the microbiota can interact via multiple mechanisms, the sequential events from exposure to pollutants to intestinal dysbiosis and the part played by the adipose tissue cannot be easily determined. Indeed, chemicals can modify the structure and activity of the microbiome but microbiota can influence the intestinal absorption and host metabolism of the chemicals highlighting the complex cross-interactions [150,152]. Nonetheless, recent studies using rodent models reported changes in SCFAs and/or BAs in response to exposure to TCDD [205], the dioxin-like PCB126 [214], the flame retardant DBPDE [107], 2-ethylhexyl diphenyl phosphate (EHDPHP) [215], BDE-47, TBBPA, BPS [212], and DEHP [208]; thus making possible that the altered adipose tissue functions could result from a direct effect of chemicals on the signaling mechanisms initiated by activation of GPR43/41 or TGR5. Further studies will have to explore whether the gut-adipose axis is directly targeted by pollutants. In addition, the way in which pollutants interfere with gonadal hormones in shaping the gut microbiota would as well be interesting to determine. Indeed, gut microbiota exhibits sexual differences probably originating from complex interactions between sex and nutrition [216]. 

## 7. Conclusions

Since the pioneering data showing that environmental pollutants can lead to dysfunctional adipocytes and contribute to obesity, a large number of publications have supported this hypothesis and proposed various redundant mechanisms of action. These mechanisms ranged from the activation of PPARγ and RXR promoting adipogenesis, and/or a large repertoire of nuclear receptor sensors for endobiotics and xenobiotics, not to mention the involvement of receptors for sex steroids and thyroid hormones, up to more sophisticated ways not strictly linked to hormonal action (not all reviewed here). It may include mitochondrial dysfunctions and oxidative changes [148], as well as alterations in the expression of circadian clock genes [149,217] epigenetic mechanisms and changes in the gut microbiota [112,150,151,152]. In addition, of major concern is maternal obesity as it is a growing risk factor for the health of the offspring and the origin of chronic diseases beyond infancy. A particular attention should be placed on the WAT metabolic programming of adipocyte size and adipogenesis throughout the development and the understanding of the full epigenetic component. Indeed, adipocyte dysfunction has been identified as a hallmark of age-related diseases, in particular cardiovascular and metabolic diseases as well as cancer.

Overall, a better understanding of the mechanisms by which environmental chemicals target adipose tissue and cause metabolic disturbances, how these mechanisms cross-interact, and the sex-specificities is essential for developing mitigating and sex-specific strategies against chemical-induced metabolic diseases. It is also essential that females are included in protocols and not only males in order to really apprehend the sex-dependent effects of those pollutants. The gut microbiota may constitute one opportunity although the modulation of gut microbiota composition through dietary intervention to improve metabolic parameters of obese patients using microbial transplantation, resveratrol supplementation or supplementation with probiotics had not been successful in all published reports [218,219,220]. Still, a recent study has opened new preventable or curative treatments of metabolic syndrome. The authors identified key microbiota metabolites that show reduced levels in metabolic syndrome conditions and succeeded in improving both dietary- and genetic-induced metabolic impairments through providing either the bacteria capable of restoring gut microbiota or the missing microbial metabolites [221]. Other strategies may reside in enhancing metabolism of the environmental pollutants through providing bacteria capable of degrading pollutants which will be particularly useful in the case of persistent chemicals as a way to accelerate their clearance. As part of the gut adipose axis, a more direct strategy may as well focus on SCFAs and BAs and the activation of G-protein-coupled receptors such as the use of semi-synthetic BAs which have been found to enhance adipose tissue beiging and thermogenesis [222] but without the hepatic side effects and gallstones formation. 

As a scenario without environmental pollutants is not a realistic scenario in current societies, new strategies must be developed to mitigate the effects induced by environmental pollutants and limit the increasing rate of diabetes and obesity observed in the world. European Union estimates that five EDCs alone add an annual cost of € 28–29 billion to obesity and diabetes-related healthcare [223]. Encouraging people to avoid the use of endocrine disruptors and promoting the substitution of harmful chemicals with hopefully harmless chemicals will not be sufficient. In a wider way and although dysfunctional adipocytes are central in obesity development and metabolic-related diseases, strategies indirectly targeting the liver and the muscle may also have beneficial consequences on adipose tissue homeostasis. Finally, we should also keep in mind that the optimal strategies as well as the best tissue to target may be different in men and women and may as well depend on the developmental period of life (infancy, adolescent, adult, elderly).

## Figures and Tables

**Table 1:** Metabolic phenotypes of male and female mice deleted (specifically in adipocytes or global KO) for various sex steroid receptors, enzymes regulating local production of active sex steroids and growth hormone receptor (or over-expressing them) as presented Section 2.3.3 and Section 2.3.4.

Gene Deleted/Over-Expressed	Metabolic Phenotype in Males	Metabolic Phenotype in Females	Ref.
**Sex steroid receptors**			
Estrogen receptor (ERα)			
Global KO	Increased body weight and fat mass, insulin resistance and impaired glucose tolerance	Increased body weight and fat mass, insulin resistance and impaired glucose tolerance	[49]
Adipose specific deletion	No changes in total body weight but enhanced expression of fibrosis markers and inflammation in the adipose tissues; no changes in glucose tolerance; protective role of ERβ against fibrosis and inflammation when ERα is lacking (although it is less important than in females deleted for ERα in adipocytes	No changes in total body weight but enhanced expression of fibrosis markers and inflammation in the adipose tissues; worsening glucose intolerance despite no change in plasma levels of 17β-estradiol; protective role of ERβ against fibrosis and inflammation when ERα is lacking	[48]
Androgen receptor (AR)			
Global AR KO or hepatic-, muscle- or pancreas-specific KO	Obesity with decreased energy expenditure, hepatic steatosis, and insulin resistance	No data	[55]
Adipose specific deletion	Do not exhibit obesity or enhanced fatness probably due to increased estradiol levels measured in adipose tissue	No data	[56]
**Enzymes regulating local production of active sex steroids**			
Aromatase (Arom)			
Global KO (ArKO)	ArKO mice grow larger with aging than age-matched wild-type mice and exhibit dramatic expansion of the visceral depot with larger adipocytes	ArKO mice grow larger with aging than age-matched wild-type mice and exhibit dramatic expansion of the visceral depot with larger adipocytes	[57]
Overexpression in adipocytes	Reduced inflammation and increased insulin sensitivity of adipose tissue	No data	[32]
Steroid sulfatase (STS)			
Overexpression in adipocytes	Worsening of metabolic health, including weight and fat gain, as well as aggravating insulin resistance, which was due to enlarged adipocytes, increased fat inflammation, and dysregulated adipogenesis. These effects were related to increased androgenic activity since castration abolished the effects.	Alleviation of metabolic functions and decreased inflammation in the adipose tissue	[61]
Estrogen sulfotransferase (EST)			
Global KO	Worsening of metabolic abnormalities under obesity conditions with highly dysfunctional adipocytes characterized by elevated expression of several macrophage markers and a high density of crown-like structures, all signs of local inflammation.	A metabolic benefit is described related to enhanced estrogenic activity in the liver; no effect on the adipose tissues.	[59]
11β-HSD1			
Overexpression in adipocytes	Hypertrophy of visceral adipose tissue, insulin resistance, diabetes, dyslipidemia, and hypertension in mice	No data	[64]
adipose specific deletion	Reduced visceral fat accumulation; higher expression of PPARγ; lower leptin and resistin levels; increased energy dissipation (UCP2)	No data	[65]
Growth hormone receptor (GhR)			
Adipose specific deletion	Improvement of with improved glucose homeostasis and decreased TG levels, inflammation and fibrosis; increased adiposity due to increased cell size in the subcutaneous compartment	Metabolic profile similar to males with subtle differences	[69]

**Table 2:** Synthesis of various endocrine-disrupting chemicals (EDCs) and some of their characteristics and adverse metabolic effects in relation with the adipose tissue as presented Section 3, Section 4, Section 5 and Section 6.

Chemicals	Examples	Degradability and Route of Exposure	Sources	Possible Mechanisms Relevant for Energy Metabolism	In Vitro Effects Potentially Described (Mostly Using 3T3-L1 Cells)	In Vivo Metabolic Effects Potentially Described on Body Weight/Fat Mass	Ref.
Dioxins and dioxin-like PCBs	2,3,7,8-tetrachlorodibenzo-p-dioxin (TCDD); coplanar PCB-77, 126	Persistent; exposure through diet (mainly)	Dioxins: industrial by-products, forest fires, volcanic eruption; DL-PCBs: industrial products in electrical capacitors	AhR; cross-interaction with PPARγ and ERs	Alteration of adipogenesis; adipocyte cell size; levels of inflammatory markers and resistance to insulin	Obesogen at low doses (≠high and acute exposure); changes in body weight and/or body fat composition in both sexes depending on period of exposure (maternal or post-weaning); effects on microbiota	[91,92,93,94,95,96,97,98,99,100,101,159,205,207,211,214]
Non-dioxin like PCBs	PCB153; PCB101; PCB180	Persistent; exposure mainly from diet	Industrial products used for their low-flammability and high conductivity properties	Estrogen and thyroid signaling; indirect effect via PXR and CAR; RXR and retinoid signaling	changes in adipocyte cell size; inflammatory markers, lipid metabolism;	Obesogen in males; Increased adipocyte differentiation; alteration of retinoid synthesis and of lipid metabolism	[11,70,95,102,104,105,158]
Organochlorine pesticides	DDT and its metabolite DDE	persistent; bioaccumulation through the food chain	agricultural pesticides	Sex steroid and thyroid hormone pathways; PPAR	Alteration of adipogenesis; enhancement of adipogenesis and induction of unhealthy mature adipocytes	Impaired metabolic homeostasis in female but not in male mice after perinatal exposure to DDT	[109,146,147,181]
Organotin compound	Tributiltyn (TBT); dibutyltin (DBT)	Persistent	Powerful biocide; marine shipping applications as fungicides; diet exposure	PPARs, RXRs, ERs	Alteration of adipocyte lineage commitment in a RXR-dependent manner; promotion of adipogenesis in a PPARγ-dependent manner but not all of the health-promoting activities induced by Pparγ resulting in unhealthy adipocytes;	Obesogen in both sexes; adipocyte lineage commitment; adipocyte differentiation; epigenetic mechanisms and transgenerational effects; effects on microbiota	[110,111,112,113,114,115,209,210]
Flame retardants	Polybrominated flame retardants (PBDEs; TBBPA; EHDPHP)	Persistent	Added to manufactured materials such as plastics, textiles to delay development of ignition; present in house dusts;	Estrogen and thyroid hormone pathways; PPARγ, GR, PXR	Lipid accumulation; enhanced adipogenesis and expression of markers of adipogenesis; enhances oxidative stress	Enhanced body weight; reduced glucose uptake; enhances the expression of inflammatory markers; triglyceride synthesis; bile secretion; effects on microbiota	[11,70,106,107,108,141,142,143,148,212,215]
Phenylsulfamides	Tolylfluanid (TF)	Not persistent	Active ingredient in fungicides and wood preservatives	GR; mitochondrial metabolism	Obesogen in vivo;	Increased adipocyte differentiation; sex-dimorphism	[130,192,193,194]
Phthalates	DEHP	Short half-life; exposure through diet and hand-to-mouth behavior in children	Plastic components, cosmetics, medical equipment	PPARs, CAR/PX, GR	exposure is associated with metabolic-related disturbances	Adipocyte differentiation; sex-dimorphism; effects on microbiota	[123,125,127,128,129,130,208,213]
Bisphenols	BPA, BPS	Short half-life; exposure through diet and water drinking because of leaching of the chemical from cans, plastic bottle.	Plastic components, cosmetics, disinfectants, thermal paper receipts	ERs and estrogen related receptors (ERRs), AR, TR, GR, PPARγ	BPA can increase metabolic disturbances within insulin-sensitive organs including the adipose tissue eventually leading to type 2 diabetes without systematically causing gain of weight; evidences for obesogenic properties of BPS	Adipocyte differentiation; Insulin sensitivity; expression of adipogenic and inflammatory markers; ex-dimorphism; effects on microbiota	[117,118,119,120,121,122,123,124,125,126,130,131,132,133,134,135,136,137,183,184,185,186,187,206,212]
